# Supplementary material for: Direct observation of charge separation in an organic light harvesting system by femtosecond time-resolved XPS
Source: Nat Commun. 2021 Feb 19;12:1196. doi: 10.1038/s41467-021-21454-3 (PMC7895929; doi:10.1038/s41467-021-21454-3)
Supplement: Supplementary file 1 — Supplementary Information [file 41467_2021_21454_MOESM1_ESM.pdf]

Supplementary Information

Direct observation of charge separation in an organic light harvesting system by femtosecond time-resolved XPS

Friedrich Roth,[1*] Mario Borgwardt[2], Lukas Wenthaus[3], Johannes Mahl[2], Steffen Palutke[4], Günter Brenner[4], Giuseppe Mercurio[5], Serguei Molodtsov[1,5,6], Wilfried Wurth[3,4,7], Oliver Gessner[2*], and Wolfgang Eberhardt[3*]

[1]Institute of Experimental Physics, TU Bergakademie Freiberg, D-09599 Freiberg, Germany

[2]Chemical Sciences Division, Lawrence Berkeley National Laboratory, Berkeley, California 94720, USA

[3]Center for Free-Electron Laser Science / DESY, D-22607 Hamburg, Germany

[4]Deutsches Elektronen-Synchrotron DESY, Notkestraße 85, 22603 Hamburg, Germany

[5]European XFEL GmbH, Holzkoppel 4, 22869, Schenefeld, Germany

[6]ITMO University, Kronverksky pr. 49, St. Petersburg, 197101, Russia

[7]Universität Hamburg, Luruper Chaussee 149, 22761, Hamburg, Germany

[*] corresponding authors, email: friedrich.roth@cfel.de, ogessner@lbl.gov, wolfgang.eberhardt@cfel.de

Supplementary Note 1. TR-XPS measurements with pristine materials

Supplementary Fig. 1 and 2 show the time-dependent C 1*s* XPS signals from the two pristine materials, C_{60} and CuPc, respectively, deposited on an n-type Si(100) substrate. Pump-pulse induced changes are limited to the formation of sidebands due to the laser-assisted photoelectric effect (LAPE) during temporal overlap between the pump and probe pulses[1], and a rigid shift of 30 meV of the entire photoelectron spectrum at positive compared to negative delays. The latter is ascribed to transient surface photovoltage effects in the Si substrate.

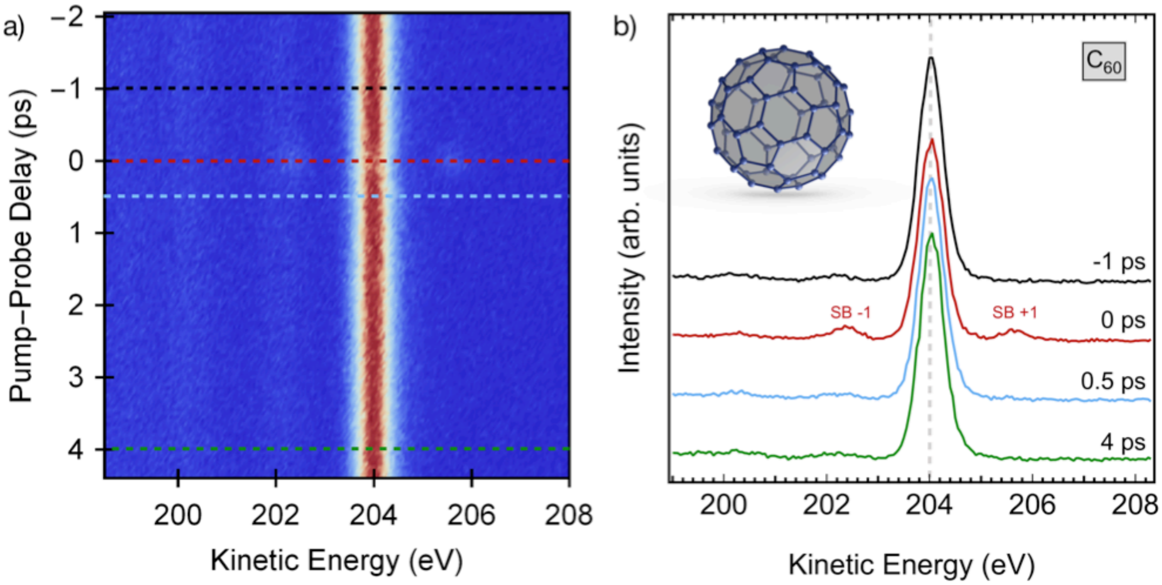

Supplementary Figure 1. Time-resolved XPS spectra of pristine C_{60}. a) 2D false-color map of the time-dependent C 1*s* signal of a thin layer of C_{60} (approx. 6 nm) on top of a pre-cleaned n-type Si wafer measured at a photon energy of 495.8 eV. Signal intensities increase from blue to red. b) Cuts at four different pump-probe delays as indicated in the false-color map.

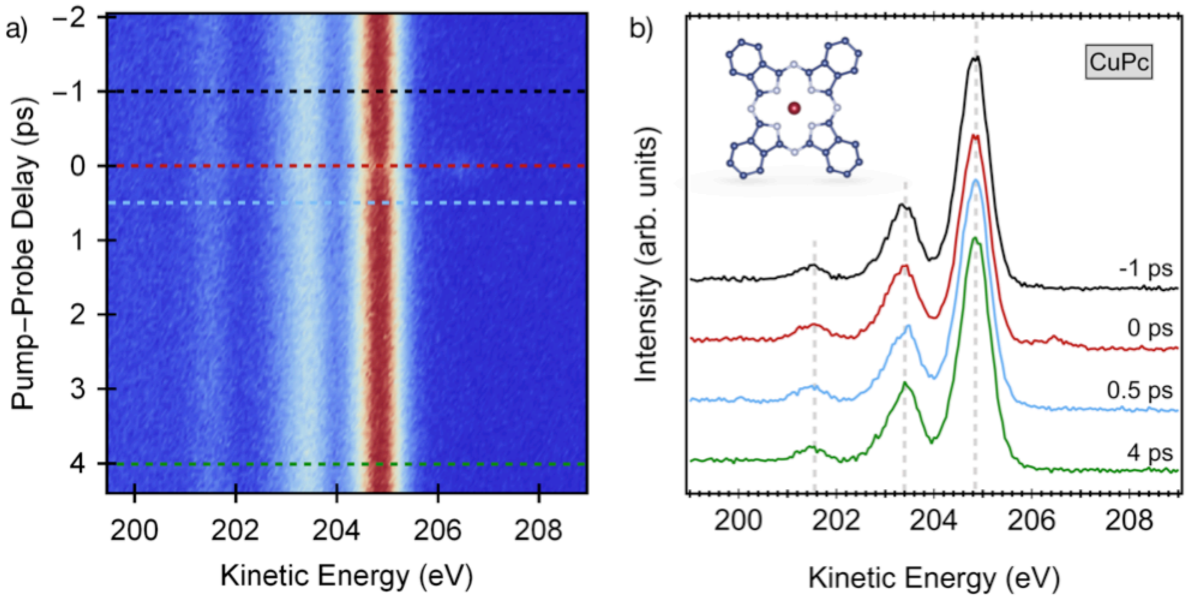

Supplementary Figure 2. Time-resolved XPS spectra of pristine CuPc. a) 2D false-color map of the time-dependent C 1*s* signal of a thin layer of CuPc (approx. 5.5 nm) on top of a pre-cleaned n-type Si wafer measured at a photon energy of 495.8 eV. Signal intensities increase from blue to red. b) Cuts at four different pump-probe delays as indicated in the false-color map.

Supplementary Note 2. Detailed description of the global fit

Time-dependent changes in the XPS spectra of the CuPc-C_{60} heterojunction are modeled based on a linear combination of the pure compound spectra (C_{60} and CuPc). A global fit procedure is applied in order to reproduce the experimental data and to analyze the dynamic trends quantitatively in the time and energy domains. The linear combination is composed of three components: two copies of the pristine C_{60} spectrum, ($C_{60}(0)$ and $C_{60}(1)$) and a single copy of the pristine CuPc spectrum. The two C_{60} components describe the spectral contributions from unperturbed C_{60} molecules (labelled $C_{60}(0)$) and from those C_{60} molecules whose photoemission is affected by the transient presence of additional, injected charges in their vicinity (labelled $C_{60}(1)$). The fit procedure is designed to determine the time-dependent kinetic energy offsets (KEOs) of the components relative to the pristine steady-state spectra, and the component amplitudes, subject to a number of boundary conditions as described in the following. The motivation behind the boundary conditions is to keep the number of free fit parameters as small as possible while achieving a good fit result. The boundary conditions are based on a set of underlying model assumptions:

1) The first assumption is that the predominant effect of interfacial charge-transfer (CT) dynamics is captured by a change in the amplitude ratio between $C_{60}(0)$ and $C_{60}(1)$, while the difference ΔE between their KEOs as well as the sum of their amplitudes, $A_{C60} = A_{C60(0)}(t) + A_{C60(1)}(t)$, remain

constant. The underlying physical picture is that core-hole screening by an injected electron is expected to induce a well-defined shift ΔE of $C_{60}(1)$ relative to $C_{60}(0)$, and that a change in the number of screening electrons in the C_{60} domain will only change the relative contributions from the two components but not ΔE or the total photoemission signal A_{C60} from all C_{60} molecules. The KEO difference ΔE and the total amplitude A_{C60} are free fit parameters.
2) The second assumption is that the KEOs of all components can be characterized by a single value before the interaction of the pump pulse with the sample, and a single value after the interaction, and that the transition between these values is described by an error function that corresponds to the instrument response function (IRF). Time-zero, as well as the temporal width of the error function are fixed to the result of the IRF calibration measurements based on the LAPE effect as described in the main manuscript. The underlying physical picture is that all spectral components may be subject to an SPV effect in the Si substrate, as described in Section I for the pristine CuPc and C_{60} films.
3) The third assumption is that the time-dependent change in the amplitude ratio between the $C_{60}(0)$ and $C_{60}(1)$ components is captured by the following model, which is schematically illustrated in Fig. 5b of the main manuscript. The population n_{ICT} of the initially excited, fast decaying ICT states and the population n_{sep} of the charge-separated electronic states, which are populated via decay of the ICT states, are described by two coupled rate equations:

$$\frac{\mathrm{d}n_{ICT}}{\mathrm{d}t} = g(t - t_0, \sigma_{IRF}) - (k_{sep} + k_{rec})n_{ICT}(t) \quad (1)$$

$$\frac{\mathrm{d}n_{sep}}{\mathrm{d}t} = k_{sep}n_{ICT}(t) - k_{src}n_{sep}(t) \quad (2)$$

Here, k_{sep} is the charge separation rate from the ICT state into a long-lived population, and k_{rec} is the recombination rate from the ICT state back to the ground state (timescales and rates are related by $\tau_i = 1/k_i$). The decay of the charge-separated population occurs via the rate k_{src}, which is fixed to $k_{src} = (280 \text{ ps})^{-1}$ based on the results of a previous picosecond time-resolved XPS study[2]. $g(t)$ indicates a Gaussian-shaped initial population function and is fixed to the measured IRF. The time-dependent amplitude $A_{C60(1)}(t)$ of $C_{60}(1)$ is assumed to be proportional to the total population of ICT and charge-separated states, $A_{C60(1)}(t) = A_g\left(n_{ICT}(t) + n_{sep}(t)\right)$, where A_g is a constant amplitude scaling factor. The two rates k_{sep} and k_{rec} as well as the scaling factor A_g are free fit parameters.
4) The amplitude A_{CuPc} of to the CuPc component is assumed to be constant for all times and is also a free fit parameter.

This global fit approach leads to a total of ten free fit parameters describing the entire energy- and time-dependent transient signal: 3 amplitudes (A_g, A_{C60}, A_{CuPc}), 2 rate constants (k_{sep}, k_{rec}), 4 KEOs ($C_{60}(0)$ before/after time zero, CuPc before/after time zero) and the KEO offset ΔE between $C_{60}(0)$ and $C_{60}(1)$. Note, however, that the dynamic evolution of the spectra for positive delays

beyond the pump-probe temporal overlap region, i.e., the key physics of the photoinduced interfacial processes, is entirely described by only 3 free fit parameters, A_g, k_{sep} and k_{rec}.

The global fit optimization was performed by minimizing the sum of the squares of the residuals when subtracting the linear combination of basis spectra from the measured transient signal. In the vicinity of time zero, the temporal overlap of the X-ray and NIR pulses leads to a time-dependent intensity redistribution within the photoelectron spectra into LAPE sidebands. To properly disentangle the system's internal photodynamics from this laser-induced effect, the simulation of the sideband formation is included in the fit procedure based on a modified LAPE response function[1,3,4]. Details will be described in a forthcoming publication[5].

Supplementary References

1. Miaja-Avila, L. *et al.* Laser-Assisted Photoelectric Effect from Surfaces. *Phys. Rev. Lett.* **97**, 113604 (2006).
2. Roth, F. *et al.* Efficient charge generation from triplet excitons in metal-organic heterojunctions. *Phys. Rev. B* **99**, 020303 (2019).
3. Saathoff, G., Miaja-Avila, L., Aeschlimann, M., Murnane, M. M. & Kapteyn, H. C. Laser-assisted photoemission from surfaces. *Phys. Rev. A* **77**, 022903 (2008).
4. Arrell, C. A. *et al.* Laser-Assisted Photoelectric Effect from Liquids. *Phys. Rev. Lett.* **117**, 143001 (2016).
5. Borgwardt *et al.*, *in preparation*.
